# A Study of Silver Decoration on Carbon Nanotubes via Ultrasonic Chemical Synthesis and Their Reinforced Copper Matrix Composites

**DOI:** 10.3390/nano13050887

**Published:** 2023-02-27

**Authors:** Dengfeng Tian, Yichun Liu, Jie Yu, Qi Zhao, Jingmei Tao, Zhong Wu, Jinfeng Zhang, Yunying Fan, Yanzhang Liu, Caiju Li, Jianhong Yi

**Affiliations:** 1 School of Materials Science and Engineering, Kunming University of Science and Technology, Kunming 650093, China; 2 Kunming Institute of Precious Metals, Kunming 650106, China; 3 School of Materials Science and Engineering, Tianjin University, Tianjin 300000, China; 4 National Experiment Teaching Demonstration Centre of Materials Science and Engineering, Tianjin University, Tianjin 300000, China; 5 Kunming Zhongtianda FRP Development Co., Ltd., Kunming 650093, China

**Keywords:** carbon nanotubes, copper matrix composites, ultrasonic chemical synthesis, mechanical properties, electrical conductivity, thermal conductivity

## Abstract

The homogeneous distribution of carbon nanotubes (CNTs) in the Cu matrix and good interfacial bonding are the key factors to obtain excellent properties of carbon nanotube-reinforced Cu-based composites (CNT/Cu). In this work, silver-modified carbon nanotubes (Ag-CNTs) were prepared by a simple, efficient and reducer-free method (ultrasonic chemical synthesis), and Ag-CNTs-reinforced copper matrix composites (Ag-CNTs/Cu) were fabricated by powder metallurgy. The dispersion and interfacial bonding of CNTs were effectively improved by Ag modification. Compared to CNTs/Cu counterparts, the properties of Ag-CNTs/Cu samples were significantly improved, with the electrical conductivity of 94.9% IACS (International Annealed Copper Standard), thermal conductivity of 416 W/m·k and tensile strength (315 MPa). The strengthening mechanisms are also discussed.

## 1. Introduction

The demand of copper-based materials in the areas of power transmission equipment, automotive manufacturing and aerospace and rail transit is moving in the direction of structural function integration, and the Cu matrix composites are of advantage with multiple integration properties. With excellent comprehensive properties, carbon nanotubes (CNTs) are considered to be the ideal one-dimensional reinforcement for Cu matrix composites [1,2,3]. However, the problems of poor spatial dispersion and poor interfacial wettability of CNTs have seriously limited their performances. Therefore, it is crucial to explore a reasonable preparation process to ensure the optimal reinforcement effect of CNTs. A variety of preparation techniques have been developed to improve the dispersion of CNTs and increase the interfacial bond strength, such as in situ synthesis [4], molecular level mixing [5], electrodeposition [1], ultrasonic spray chemical plating [6], etc. Liu et al. [7] improved the wettability and interfacial bonding between CNTs and the Cu matrix by first ball milling CNTs with titanium powder and then forming TiC on the surface of CNTs through pressureless sintering, thereby preparing high-strength and high ductility TiC-CNTs/Cu composites. Wei et al. [1] improved the dispersibility of CNTs by modifying CNTs with Ni nanoparticles. The high strength CNTs/Cu foil was prepared by electrodeposition. Yang et al. [8] prepared CNTs-Ag composite powders by the pre-discharge method and CNTs-Ag/Cu was prepared by the ball milling method to achieve synergistic strengthening of tensile strength and elongation while the composite still maintains high electrical conductivity. All these studies to improve the dispersion of CNTs in the matrix face problems, such as high cost and complicated operation. Therefore, the development of a simple and efficient preparation process route was urgently needed to realize the structural function integration of CNTs/Cu composites.

In this work, copper matrix composites reinforced with Ag-modified CNTs were prepared via ultrasonic chemical (UC) synthesis and Spark Plasma Sintering (SPS) with the expectation to obtain homogenous reinforcement distribution and better interface bonding for the purpose of the improvement of electrical conductivity, thermal conductivity and mechanical properties.

## 2. Experimental Procedures

### 2.1. Synthesis of Ag-CNTs Powders

CNTs (inner diameter from 5–12 nm, outer diameter from 20–50 nm, length from 10–20 μm) with a pureness of 99.5% were purchased from the Chinese Academy of Sciences (Beijing, China). For the pretreatment of CNTs, including acidifying, sensitizing and activating [9], firstly, the pristine CNTs were acidified (reaction temperature: 353 K, reaction time: 4 h) by adding mixed acid (V(HNO_3_): V(H_2_SO_4_):1:3). Secondly, the acidified CNTs were sensitized (SnCl_2_·2H_2_O (10 g/L) + HCl (40 g/L)) and activated (PbCl_2_ (1 g/L) + HCl (100 ml/L)) sequentially to provide active sites for chemical silver plating. Each of these pre-treatment steps was followed by filtration, washing and vacuum drying.

The CNTs suspension of 1 g/L was obtained by placing 0.1 g of pretreated CNTs into a beaker and adding ultrapure water to the beaker followed by sonication for 30 min. Then, 2 g/L of silver ammonia solution and 0.5 g/L of polyvinyl alcohol (PVA, Aladdin Bio-Chem Technology Co., Ltd., Shanghai, China) solution were added. The ultrasonic dispersion was repeated for 30 min using a PVA solution as protection. The H radicals with reducing ability was decomposed from water by providing energy through ultrasound without a reducing agent [10], which could reduce Ag^+^ in the solution into nano-Ag monomers and coat the surface of CNTs to obtain Ag-CNTs composite powders. After the reaction, the solution was loaded into centrifuge tubes using a high-speed centrifuge at 6000 r/min for 5 min and repeated five times, and finally dried in a 323 K vacuum oven to obtain Ag-CNTs powder.

### 2.2. Preparation of Ag-CNTs/Cu Composite 

**Figure 1:**
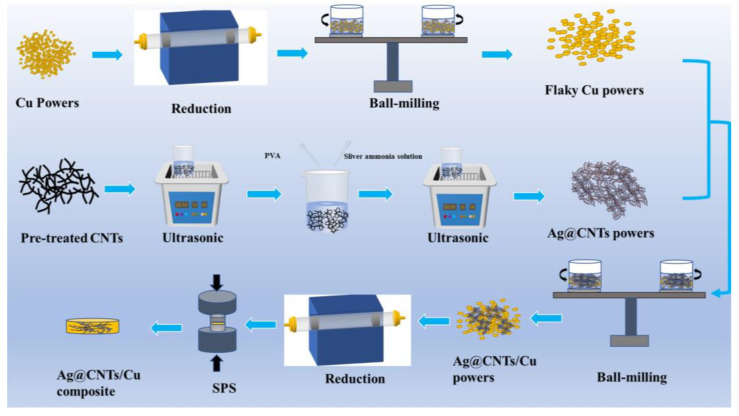
Diagram of the preparation process of Ag-CNTs/Cu composites.

The flake copper powder was obtained by ball milling in a planetary ball mill for 10 h (medium: anhydrous ethanol; ball-to-power ratio: 10:1; speed: 300 r/min). Next, the Ag-CNTs powders (M_Ag-CNTs_:M_CNTs_ = 1.15:1) were ultrasonically dispersed in ethanol for 30 min and then ball-milled together with the flake Cu powders for 3 h (ball-to-power ratio: 10:1; rotation rate: 180 r/min). After filtration and drying, the Ag-CNTs/Cu powder was obtained by reduction under the atmosphere of Ar-5%H_2_ (temperature: 300 °C, holding time 5 h). Then, 15 g of Ag-CNTs/Cu composite powder was placed in a graphite mill with a diameter of 30 mm. The mill was then placed in the SPS apparatus for sintering (temperature program: firstly, heating at 100 °C/min to 650 °C, secondly, heating at 50 °C/min to 750 °C and finally holding for 5 min before cooling naturally to room temperature, axial pressure: 50 MPa). The Ag-CNTs/Cu composites were obtained by sanding the samples on sandpaper. Figure 1 shows the flow chart of the preparation of Ag-CNTs/Cu composites. Cu and CNTs/Cu composites were prepared by the same method.

### 2.3. Characterization

The X-ray diffractometer was used to characterize the phase composition with a CuKα (λ = 0.15406 nm) radiation source and a 2θ test angle of between 10 and 90° (XRD, RIGAKU D/Max-2550, Tokyo, Japan). Raman spectroscopy was used to test the structural integrity and charge transfer of carbon materials in the sample (LABRAM HR800, HORIBA, Paris, France). X-ray photoelectron spectrometry was performed to investigate the chemical bonding state of the samples (XPS, Kratos Axis Ultra DLD, Manchester, UK). The morphologies of specimens were investigated by a field emission scanning electron microscope (FE-SEM, Nova Nano-450, FEI, Hillsboro, OR, USA). The microstructure and interfacial characteristics of the samples were characterized using a transmission electron microscopy (TEM, FEI Tecnai G2-TF30 S-Twin, Portland, OR, USA). The mechanical strength was obtained by cutting the composite material into a sample size of 18 × 2 × 1.8 mm on a universal testing machine with a loading rate of 0.2 mm/min (AG-X-100kN, SHIMADZU, Kyoto, Japan). The electrical conductivity of the samples was measured by an eddy current conductivity meter (Sigma 2008B, Xiamen Tianyan Instrument Co., Ltd., Xiamen, China). The thermal diffusion coefficients of samples were measured by the laser flash apparatus (LFA 467, NETZSCH, Luxembourg, Germany). The samples were coated with graphite on the front and back to increase the laser emissivity on the pre-absorption surface and the emissivity on the post-absorption surface. To ensure the accuracy of the data, the sample tests were repeated three times to obtain the average value. The thermal conductivity of the samples were calculated by the equation $K=\rho c_{p\;}\alpha$ [11], where K is thermal conductivity, ρ is experimental density, $c_{p\;}$ is specific heat and α is thermal diffusivity.

## 3. Results and Discussion

### 3.1. Morphology and Microstructure of Ag-CNTs and Ag-CNTs/Cu

**Figure 2:**
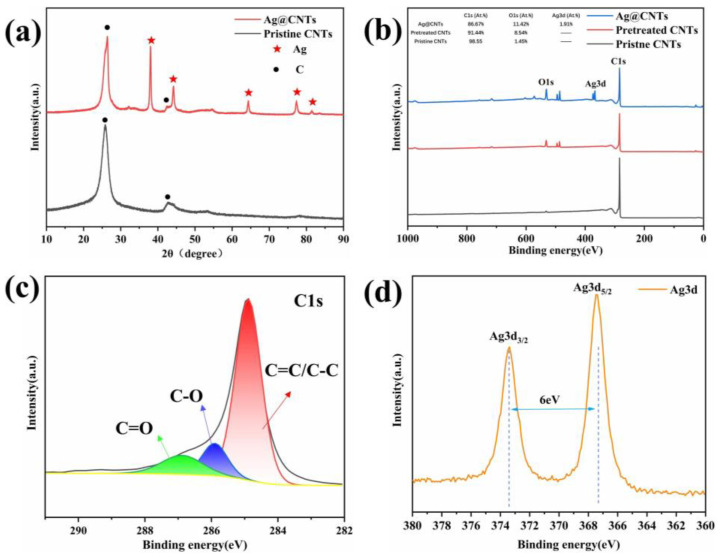
(**a**) XRD patterns of pristine CNTs and Ag-CNTs; (**b**) XPS spectra of pristine CNTs, pretreated CNTs and Ag-CNTs; (**c**) XPS C1s spectrum of Ag-CNTs; (**d**) XPS Ag3d spectra of Ag-CNTs.

The XRD patterns of the pristine CNTs and the Ag-CNTs are shown in Figure 2a. The pristine CNTs showed distinct diffraction peaks at 2θ = 26.6° and 42.5°, which coincide with the (002) and (100) crystal planes of C, respectively. The Ag-CNTs of peaks at 38.1°, 44.2°, 66.4° and 77.4° coincide with the (111), (200), (220) and (311) incidence planes of Ag (JCPDS04-0783), respectively. It was also observed that the XRD corresponding peaks of Ag were relatively sharp, indicating good crystallinity of Ag. Figure 2b shows the X-ray photoelectron spectra (XPS) of pristine CNTs, pretreated CNTs and Ag-CNTs powders. The C1s atomic percentage of the pristine CNTs was 98.55% and the O1s atomic ratio was 1.45%, while the C1s and O1s atomic percentages of the pretreated CNTs were 91.44% and 8.54%, respectively. The increase in the percentage of O1s atoms after pretreatment indicated the successful introduction of oxygen-containing groups (–OH, –COOH) [12]. The XPS spectra of Ag-CNTs powders also confirmed the presence of C and Ag elements with atomic percentages of 88.67% and 1.91%, respectively (Figure 2b). The C1s spectra of Ag-CNTs identify the presence of C=C/C–C (~284.59 eV), C–O (~285.50 eV) and C=O (~286.98 eV) (Figure 2c) [13,14,15]. In addition, Figure 2d shows the bimodal spectrum of XPS Ag3d of Ag-CNTs composite powder. The peaks of Ag3d_3/2_ and Ag3d_5/2_ were found at 373.38 eV and 367.38 eV, respectively, with a separation energy of 6 eV, which is consistent with Ag. There was also evidence of Ag being present on the surface of CNTs [16]. In summary, it can be demonstrated that Ag-CNTs composite powders can be prepared by UC without any reducing agent.

**Figure 3:**
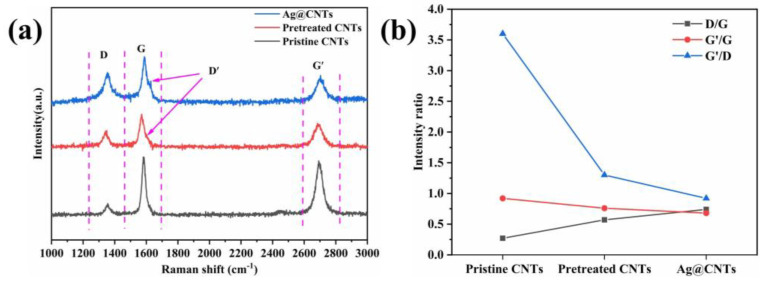
(**a**) Raman spectra of the pristine CNTs, pretreated CNTs and Ag-CNTs; (**b**) I_D_/I_G_, I_Gʹ_/I_G_, I_Gʹ_/I_D_ intensity ratios of the pristine CNTs, pretreated CNTs and Ag-CNTs.

Figure 3 shows the Raman data of pristine CNTs, pretreated CNTs and Ag-CNTs. The D peak indicates the structural integrity of the carbon material, the G peak indicates the degree of graphitization of the carbon material and the Gʹ peak is a multiplicative peak of D, which indicates the vibrational modes of the two photonic lattices [17]. The Dʹ peak of pretreated CNTs and Ag-CNTs has a certain degree of broadening at between 1600 and 1620 cm^−1^, which was caused by the defects in the tube walls of pretreated CNTs and the modification of Ag nanoparticles [18]. I_D_/I_G_ was expressed as the level of structural defects in CNTs [19]; additionally, we also assessed the relative intensity of Gʹ/G, Gʹ/D (Figure 3b). The I_Gʹ_/I_G_ was related to the graphite order extension scale and crystallinity, and I_Gʹ_/I_D_ was related to the graphite order extension and crystallinity [20]. The I_D_/I_G_ values were 0.27 for pristine CNTs and 0.57 for pretreated CNTs, indicating that the pre-treatment process added defects to the CNTs. The I_Gʹ_/I_G_ and I_Gʹ_/I_D_ values of pretreated CNTs and Ag-CNTs showed a decrease relative to pristine CNTs, which was due to more dense lattice defects and encapsulation of Ag nanoparticles in CNTs [20,21].

**Figure 4:**
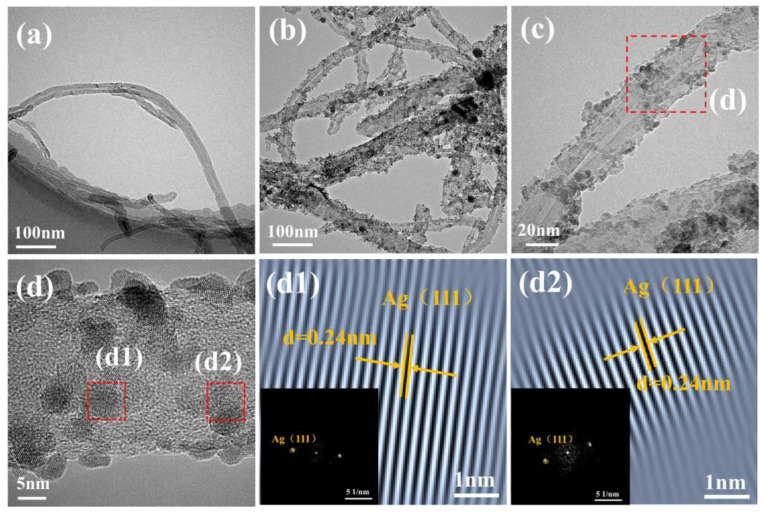
(**a**) TEM images of pristine CNTs; (**b**,**c**) TEM images of Ag-CNTs; (**d**) HRTEM images of Ag-CNTs; (**d1**,**d2**) FFT and IFFT images of the red dashed box in Figure 4d.

The TEM images of the pristine CNTs and the Ag-CNTs are shown in Figure 4. Numerous Ag nanoparticles were deposited in the tube wall of the CNTs without obvious aggregation, as shown in Figure 4b,c. Furthermore, some distortion of the wall of CNTs containing Ag nanoparticles was observed, indicating that the Ag nanoparticles were not just simply deposited on the surfaces of CNTs. The red dashed box was selected for Fast Fourier Transformation (FFT) and Inverse FFT (IFFT), Figure 4d1,d2 in Figure 4d. It can be seen that the crystal plane spacing of the diffraction spot is 0.24 nm, corresponding to the (111) crystal planes of Ag. To demonstrate the advantages of ultrasonic chemical synthesis methods in size uniformity and coating integrity of Ag nanoparticles on the surface of CNTs. The common Ag-CNTs were prepared by conventional chemical plating (CP) methods (Figure S1). Detailed fabrication processes, microstructures and the common Ag-CNTs were discussed and are provided in the Supplementary Materials.

**Figure 5:**
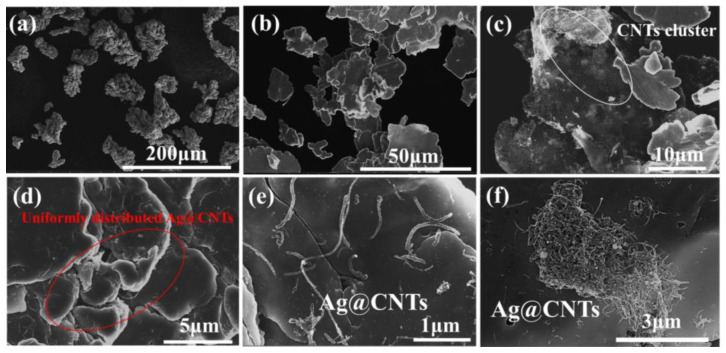
SEM images of powders: (**a**) pristine Cu powders; (**b**) flake Cu powders (after 10 h ball milling); (**c**) CNTs/Cu powders; (**d**,**e**) 0.9 vol.% Ag-CNTs/Cu powders; (**f**) 1.3 vol.% Ag-CNTs/Cu powders.

In this study, electrolytic Cu powder was used as the starting material (Figure 5a). The flaky Cu powers were obtained by controlling the ball milling process (Figure 5b). Compared to the pristine Cu powders, it had a larger surface area and better shape similarity, allowing CNTs to anchor more easily to the Cu powders surface [22,23]. Compared with CNTs/Cu powders, it can be clearly seen that Ag-CNTs in Ag-CNTs/Cu powder are uniformly adsorbed and immobilized on the surface of flaky Cu. This was attributed to the reduced mass difference between Ag-CNTs and Cu powders, which improved the dispersion of CNTs in Cu powders [24]. However, CNTs were clustered when the Ag-CNTs content was 1.3 vol.% (Figure 5f). This reduces the properties of the composites [25].

**Figure 6:**
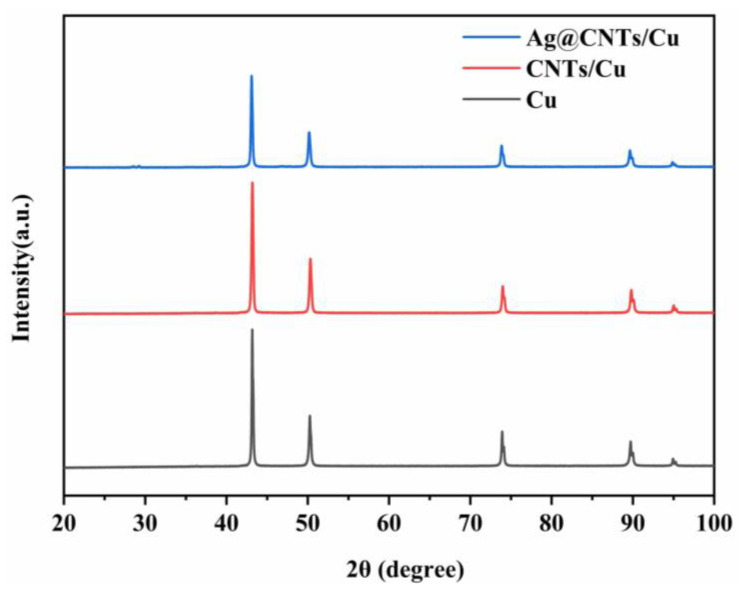
XRD spectra of Cu, CNTs/Cu and Ag-CNTs/Cu powders following reduction.

The five peaks in Figure 6 represent the (111), (200), (220), (311) and (222) crystal planes of Cu, respectively. There were no obvious peaks of C, Ag and Cu oxides (CuO, CuO_2_) found in the XRD patterns, which may be due to their content below the XRD detection limit [26]. Therefore, we assume that most of the Cu oxides were reduced to Cu.

**Figure 7:**
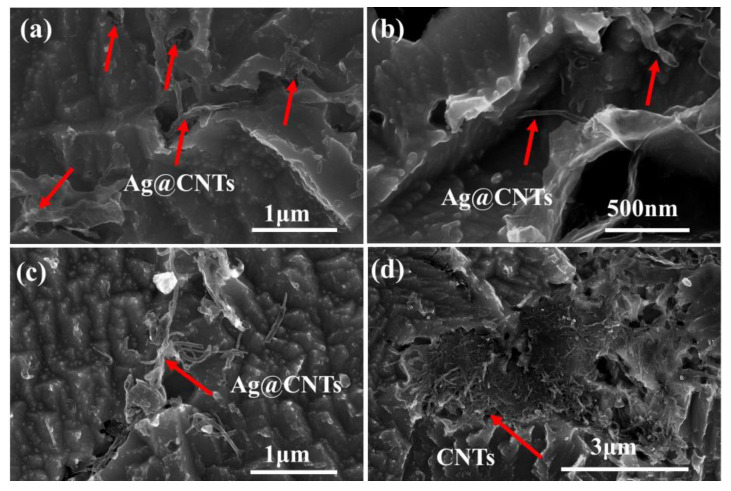
Morphology of the composites after etching: (**a**,**b**) 0.9 vol.% Ag-CNTs/Cu; (**c**) 1.3 vol.% Ag-CNTs/Cu; (**d**) CNTs/Cu.

Figure 7a,b shows the morphology of the Ag-CNTs/Cu composite after etching. It could be observed that the CNTs were randomly arranged in the etched pits of the Cu matrix, and these CNTs were uniformly distributed in the Cu matrix. The agglomerated Ag-CNTs appeared in the composites (Ag-CNTs content is 1.3 vol.%) as it was difficult for the excessive Ag-CNTs to be uniformly distributed in the Cu matrix. Then, lamellar cross-linked CNT aggregates were formed to reduce the densities of the composites, thus, reducing their overall performance [8]. The morphology of the CNTs/Cu composite is shown in Figure 7d. It was observed clearly that CNTs exhibited severe agglomeration in the Cu matrix, which also led to a deterioration in their overall properties.

### 3.2. Characterizations Microstructure of Ag-CNTs/Cu Composites

**Figure 8:**
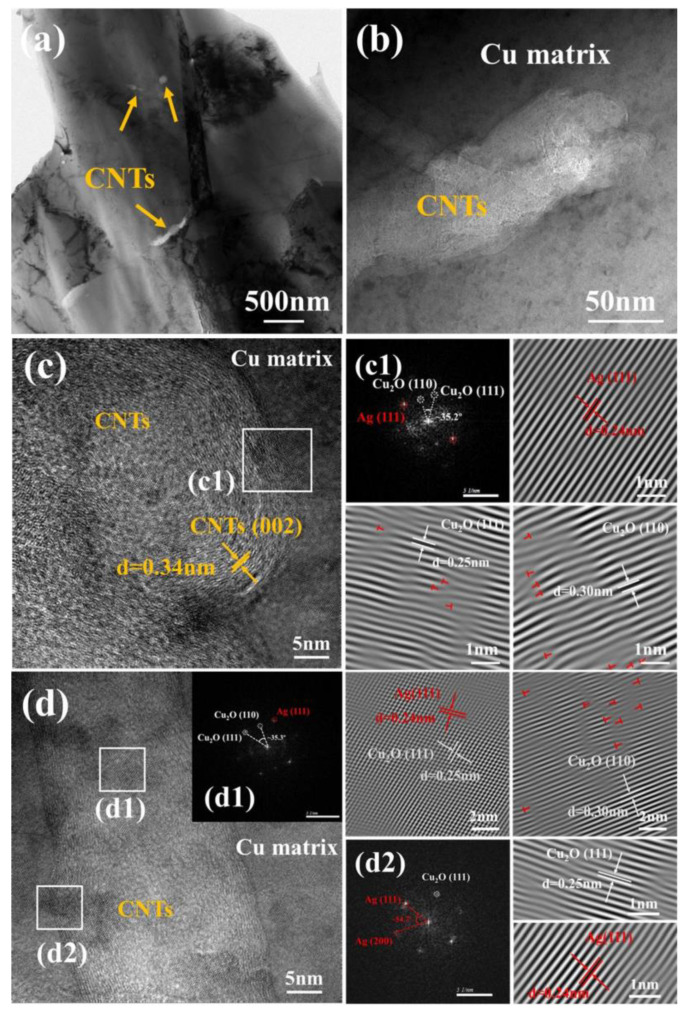
(**a**,**b**) TEM images of 0.9 vol.% Ag-CNTs/Cu composite; (**c**,**d**) HRTEM images of Ag-CNTs/Cu composites; (**c1**–**d2**) FFT and IFFT images corresponding to the white boxed areas of (**c**,**d**), respectively.

Microstructural and interfacial characters of the Ag-CNTs composite were further detected by TEM analysis. There were no obvious cracks and voids in the Cu matrix, which indicated that the Ag-CNTs/Cu composite has good interface adhesion (Figure 8). The wall gap of CNTs can be clearly seen in Figure 8c, and the crystallographic spacing was 0.34 nm, which corresponded to the (002) crystallographic plane of C. It showed that the complete structure of CNTs with high crystallinity was not destroyed during the SPS process. It could be seen by IFFT that the diffraction spots had a crystal plane spacing of 0.24 nm for the diffraction spots of Ag, which corresponded to the (111) crystal planes of Ag (Figure 8c1). The presence of Ag diffraction spots indicated that the Ag nanoparticles were not dislodged from the surface of the CNTs during the ball milling process. In addition, diffraction spots were also found with a crystal plane spacing of 0.25 nm and 0.30 nm by IFFT, which were identified as diffraction spots of Cu_2_O, corresponding to the (111) and (110) crystal planes of Cu_2_O, respectively (Figure 8c1,d1). However, the whole sintering environment was carried out under vacuum conditions, and the presence of Cu_2_O may be attributed to the binding of oxygen-containing functional groups on the surface of pretreated CNTs to the Cu matrix [26]. These Ag and Cu_2_O nanoparticles can act like “rivets” to bind the CNTs tightly to the matrix. Diffraction points of Ag and Cu_2_O were observed in the FFT images, indicating the presence of Ag–Cu coexistence regions on the surface and interface regions of CNTs (Figure 8c,d). Because of the difference of thermal expansion coefficients among CNTs and Cu, which generates thermal mismatch stresses in and around the interface region during sintering and forms dislocations, a large number of dislocations (red "T" shape) could be seen in the IFFT images [27]. Furthermore, Ag nanoparticles on the table of CNTs can enhance the mechanical properties of the composites by impeding dislocation displacement [27]. Finally, the presence of the Ag–Cu coexistence zone and Cu_2_O at the interface aided in enhancing the interfacial bonding strength of the CNTs and the Cu matrix [28].

### 3.3. Electrical and Thermal Conductivities of Composites

**Figure 9:**
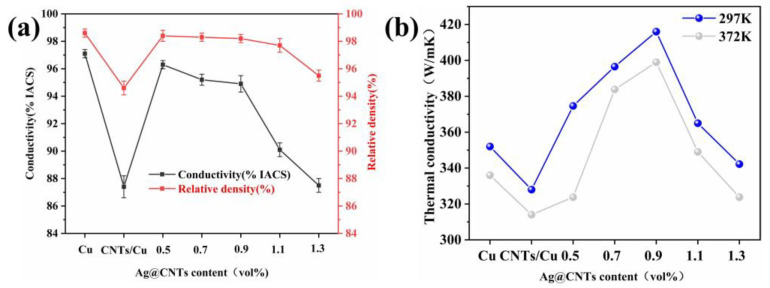
(**a**) Electrical conductivity/density; (**b**) thermal conductivity.

In this work, Ag-CNTs have been used as a reinforcing material for the incorporation into the Cu matrix. Compared with CNTs/Cu composites (87.4% IACS), the electrical conductivity of Ag-CNTs/Cu composites (87.5–96.3% IACS) showed an increase (Figure 9a). For the 1.1 vol% Ag-CNTs/Cu composite, a steep decline was shown, which was attributed to Ag-CNTs agglomeration. The electrical conductivity of the 0.9 vol% Ag-CNTs/Cu was 94.9 % IACS, 8.6% higher than that of the CNTs/Cu composite. The better interfacial bonding in Ag-CNTs composites is more profitable for electron scattering, so their conductivity was higher than that of CNTs/Cu [8]. In addition, Ag nanoparticles play an essential role in enhancing the electrical conductivity of the composites by filling and interconnecting the empty regions of the Cu matrix.

The thermal conductivity values of Ag-CNTs/Cu composites were significantly better than Cu and CNTs/Cu composites (Figure 9b). At 297 K, the thermal conductivity of 0.9 vol% Ag-CNTs/Cu composites (416 W/m·k) increased by 18.2% and 26.8% compared to Cu (352W/m·k) and CNTs/Cu composites (328 W/m·k), respectively. Within a certain range of Ag-CNTs volume fraction, it tended to rise with increasing Ag-CNTs volume fraction. It was mainly related to the CNTs alignment for CNTs/Cu, CNTs distribution and the interfacial thermal resistance (ITR) of the CNTs and Cu matrix [29,30,31,32]. First, taking into account the orientation of the distribution of CNTs in the Cu matrix, the thermal conductivity along the longitudinal axis is almost two orders of magnitude higher than that in the perpendicular axis [29,31]. Second, the homogeneous dispersion of the CNTs in the Cu matrix is a key determinant of their thermal conductivity. The homogeneous dispersion of the CNTs improved not just the density of the composite, but also enhanced the average random range of phase transitions [32]. The Ag-CNTs improved the interfacial bonding with the Cu matrix, whereas the CNTs was uniformly distributed in different directions in the Cu matrix. In this way, the Ag-CNTs/Cu composite improved the thermal conductivity. However, in CNTs/Cu composites, the severe aggregation of CNTs makes the ITR increase, so the thermal conductivity of CNTs/Cu composites was lower than Cu. Similarly, the increase in grain boundaries and ITR in Ag-CNTs/Cu composites led to a decrease in the thermal conductivity of the composites. In addition, the clustering of CNTs led to a decrease in the density of the compact composite, which led to a sharp decrease in the thermal conductivity. The thermal conductivity of the composite was also found to be higher at 297 K than at 392 K in Figure 9b. This results from the fact that the value of thermal conductivity of CNTs composites depends on the phonon and electron transport in the Cu matrix of CNTs, and the presence of interfaces and high temperatures will lead to a significant decrease in their phonon thermal conductivity [33,34].

### 3.4. Tensile Strength of Composites

**Figure 10:**
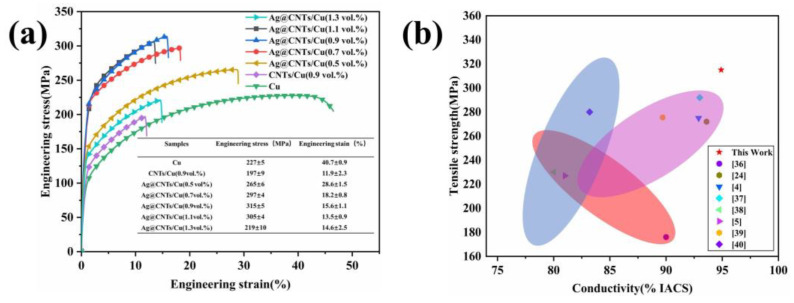
(**a**) Stress–strain curves; (**b**) comparison of tensile strength and electrical conductivity of the CNTs/Cu composites between this work and the other literature [4,5,24,36,37,38,39,40].

Figure 10 shows the stress–strain curves of Ag-CNTs/Cu composites with various volume fractions of Ag-CNTs and comparison samples. The tensile strength of the composites shows a significant trend with increasing volume fraction of Ag-CNTs in a certain volume fraction range, while the tensile strength of the 0.9 vol.% Ag-CNTs/Cu reached a maximum (315 MPa). It was raised by 38.8% and 59.9% compared to Cu and CNTs/Cu, respectively. In CNTs/Cu composites, the CNTs are mechanically bonded to the Cu matrix with a very weak bond, resulting in lower strength and elongation than Cu [35]. Similarly, we found that the tensile strength and elongation of the Ag-CNTs/Cu composite decreased when the volume fraction of Ag-CNTs was 1.1 vol.%. Figure 10b compares the tensile strength and electrical conductivity of the CNTs/Cu composites in this work with the other literature. The Ag-CNTs/Cu composites prepared in this work have significant advantages in terms of tensile properties and electrical conductivity.

**Figure 11:**
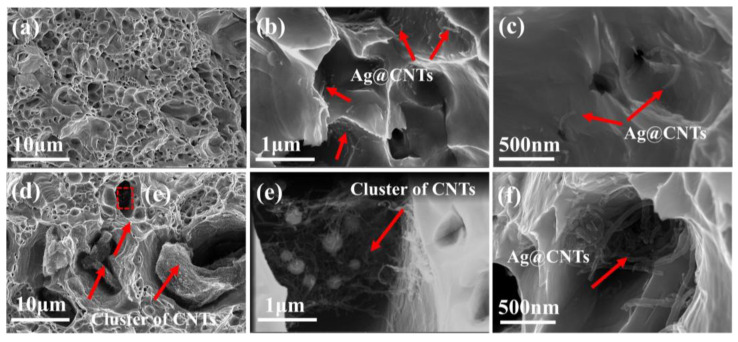
Fracture morphology of the composites: (**a**–**c**) 0.9 vol.% Ag-CNTs/Cu; (**d**,**e**) CNTs/Cu; (**f**) 1.3 vol.% Ag-CNTs/Cu morphology of the composites.

Figure 11 shows the fracture surfaces of composites. In the fracture morphology of Ag-CNTs/Cu (0.9 vol.%) composites we can find a great number of ligaments, where the surface underwent plastic deformation before fracture failure (Figure 11a). The CNTs exposed in the ligaments can be seen in the tough fossa, in addition to the CNTs that were pulled out in Figure 11c, and were found to be smaller in diameter in the exposed portion. It indicated that the Ag-CNTs were strongly bonded to the Cu matrix and formed strong interfacial bonds, which acted as a load transfer in the tensile process and provided higher strengthening efficiency [4]. The CNTs clusters caused the formation of cavities and fractures in the CNTs/Cu composite, which led to the deterioration of tensile properties (Figure 11c,d) [41]. Similarly, Ag-CNTs/Cu (1.3 vol.%) showed a great number of Ag-CNTs clusters in ligaments. This leads to stress concentration in the composite, forming a crack source, and external loading leads to crack extension during the tensile process. In other words, failure fracture occurs at low stresses with reduced strength and strain [8].

### 3.5. Strengthening Mechanism

The strengthening mechanisms of the Ag-CNTs/Cu composite include grain refinement [42], Orowan strengthening and load transfer strengthening [43], which can be expressed as follows:

(1) $$\sigma c=\sigma_{m}+\Delta \sigma_{GR}+\Delta \sigma_{Orowan}+\Delta \sigma_{L.T}+\Delta \sigma_{T.M}$$

where σc is the yield strength of Ag-CNTs/Cu composites, $\sigma_{m}\;$ is the yield strength of Cu, $\Delta \sigma_{GR}$ is the grain refinement enhancement contribution, $\Delta \sigma_{Orowan}$ is the Orowan reinforcement contribution, $\Delta \sigma_{L.T}$ is the load transfer enhancement contribution and $\Delta \sigma_{T.M}$ is the thermal mismatch enhancement contribution. The thermal mismatch effect caused by the different coefficients of thermal expansion (CTE) of CNTs and the Cu matrix in the CNTs/Cu composite was small and, therefore, negligible [12].

In the composite, the uniformly dispersed CNTs could act as a barrier to grain boundary migration. The addition of CNTs could, therefore, play a role in the refinement of the grains; the more refinement, the more grain boundaries. The amount of modifying grain refinement added to the strength of the composite can be calculated using the Hall–Petch equation [44]:

(2) $$\;\;\Delta \sigma_{GR\;}=K\left(dc^{-\frac{1}{2}}-d0^{-\frac{1}{2}}\right)$$

where K is a constant (0.07 MPa $m^{-\frac{1}{2}}$) [45], dc is the average size of the Ag-CNT/Cu composite and d0 is the average size of Cu. From the om images (Figure S2), it could be known that dc = 4.65 μm, d0 = 7.59 μm, and $\Delta \sigma_{GR\;}$ is calculated to be 7.1 MPa.

CNTs reinforcement in the Cu matrix could impede dislocation movement and form dislocation loops and plugging dislocations, thus acting as Orowan strengthening. It could be calculated using the Orowan–Ashby equation [12].

(3) $$\Delta \sigma_{Orowan}=\frac{0.13Gb}{\lambda }\ln\frac{dp}{2b}$$

Among them, G is the modulus of elasticity of Cu (42.1 GPa); b is the Burger vector of the Cu matrix (b = 0.256 nm); λ ($\lambda =dp\left(\sqrt{\frac{\pi }{2vf}}-1\right)$) is the active spacing of adjacent CNTs; $dp=\sqrt[{3}]{\frac{3dl}{2}}$, d (45 nm) and l (15 µm) represent the diameter and mean length of CNTs, respectively; vf (0.9%) is the percentage by volume Ag-CNTs. $\Delta \sigma_{Orowan}$ is calculated to be 20.5 MPa.

$\Delta \sigma_{L.T}\;$ can be calculated by the shear hysteresis model proposed by Kelly and Tyson [46].

(4) $$\Delta \sigma_{L.T}=Vf\sigma f\left(1-\frac{lc}{2l}\right)l+\sigma m\left(1-vf\right)-\sigma m\left(l\geq lc\right)$$

(5) $$\Delta \sigma_{L.T}=Vf\left(\frac{\tau y}{d}\right)l+\sigma m\left(1-vf\right)-\sigma m\left(l\leq lc\right)$$

(6) $$lc=\frac{df\sigma f}{2\tau y}\;\;\;\;\;\;\;\;\tau y\approx \frac{\sigma_{my}}{2}$$

Among them, σf is the tensile strength of CNTs (σf=30Gpa) [24], lc is the critical length of CNTs, d (45 nm) and l (15 μm) are the diameter and length of CNTs, respectively, and it is calculated that lc = 23.3 μm. Since l ˂ lc, formula (5) is chosen for calculation. $\sigma_{m}$ (58 MPa) is the yield strength of Cu and  τy (29 MPa) is the interfacial shear stress along the surface of the reinforcement. It is calculated that $\Delta \sigma_{L.T\;}$= 86.5 MPa.

**Figure 12:**
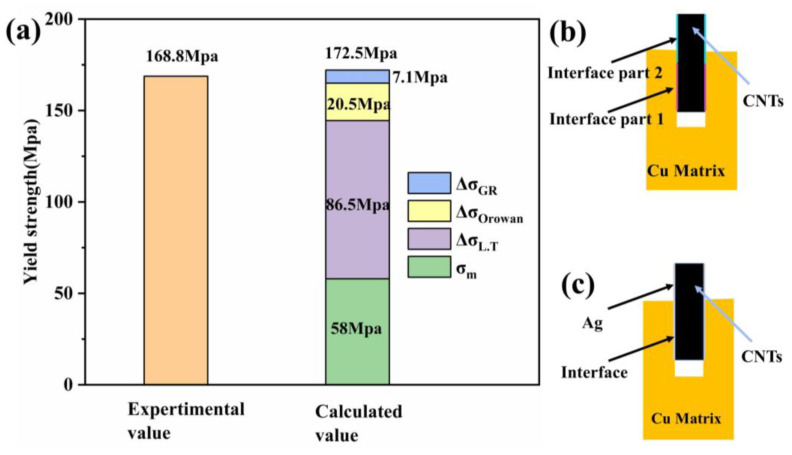
Comparison of calculated yield strength values of composites with their experimental values (**a**); interface analysis of pullout force of CNTs/Cu (**b**) and Ag-CNTs/Cu (**c**).

Figure 12a shows the comparison of each contribution value between the experimental yield strength (168.8 MPa) and the theoretical yield strength (172.5 MPa) of the Ag-CNTs/Cu composite. The calculated yield strength is larger than the experimental value, and by comparing the three strengthening mechanisms, the main strengthening mechanism for Ag-CNTs/Cu composites is load transfer strengthening ($\Delta \sigma_{L.T}\;$= 86.5 MPa). This is due to the perfect interfacial bonding of the CNTs to the Cu matrix and due to alignment of CNTs along direction of tensile force. However, the dispersal and interfacial combination of CNTs in the Cu matrix could only be improved to a mobile extent. Figure 12b,c shows the pull-out force interface analysis of CNTs/Cu and the Ag-CNTs/Cu composite. The interfacial region of CNTs and Cu of CNTs/Cu composites could be divided into two parts: the embedding region (interface part 1) and the pull-out region (interface part 2), which repeatedly failed and were offset by the interaction [46]. However, for the Ag-CNTs/Cu composite, the Ag-CNTs contribute to the entire interface during the pull-out process and their tensile strength increases with the CNTs length. Therefore, Ag-CNTs/Cu has a tensile strength higher than CNTs/Cu.

## 4. Conclusions

In this work, we prepared Ag-CNTs by an efficient and clean method (UC), and Ag-CNTs/Cu composites with excellent properties were obtained by powder metallurgy. The specific conclusions are as follows:

1. Uniform Ag nanoparticles were deposited on the surface of CNTs by ultrasonic chemical synthesis;

2. Ag-CNTs improve the dispersion and interfacial bonding of CNTs in the Cu matrix; hence, the Ag-CNTs/Cu composites have excellent tensile strength (315 MPa) and thermal conductivity (416 W/m·k) and maintain a high electrical conductivity (94.9% IACS);

3. The main strengthening mechanism of Ag-CNTs/Cu was calculated to be load transfer strengthening (68.5 MPa).

In conclusion, this method provides a new concept for solving the problems of poor dispersion and interfacial bonding of CNTs in copper matrix composites.

## Data Availability

The datasets used or analyzed during the current study are available from the corresponding author upon reasonable request.

## Supplementary Materials

The following supporting information can be downloaded at: https://www.mdpi.com/article/10.3390/nano13050887/s1, Figure S1: TEM images of Ag-CNTs powders prepared by CP at different magnifications; Figure S2: OM images and average grain size of the composites: (a) pure Cu; (b) CNTs/Cu; (c) 0.9 vol.% Ag-CNTs/Cu. The References [6,47,48] are cited in the Supplementary Materials.
