# Supplementary material for: A Study of Silver Decoration on Carbon Nanotubes via Ultrasonic Chemical Synthesis and Their Reinforced Copper Matrix Composites
Source: Nanomaterials (Basel). 2023 Feb 27;13(5):887. doi: 10.3390/nano13050887 (PMC10005354; doi:10.3390/nano13050887)
Supplement: Supplementary file 1 [file nanomaterials-13-00887-s001.zip › nanomaterials-2228034-Supplementary.pdf]

# Supplementary Materials

# A Study of Silver Decoration on Carbon Nanotubes via Ultrasonic Chemical Synthesis and Their Reinforced Copper Matrix Composites

## 1. Morphology of Ag-CNTs prepared by chemical plating

Preparation of Ag-CNT powders with reference to the methods used in the literature [1]. Figure S1 shows that the Ag-CNT powders were prepared by the chemical plating (CP) method. The presence of Ag particles of widely varying sizes and shapes on the surface of CNTs were clearly visible. Meanwhile, a small amount of Ag nanoparticles was observed on the surface of CNTs, as shown in Figure S1b. For that case, inhomogeneous coatings on CNTs and overgrown Ag nanoparticles were usually the result, as reported in Ref. [2,3]. Correspondingly, upon comparison, it is found that Ag-CNTs prepared by ultrasonic chemical plating have the advantage of being more homogeneous and convenient for plating than CP, as well as being low-cost and free from contamination without the use of reducing agents.

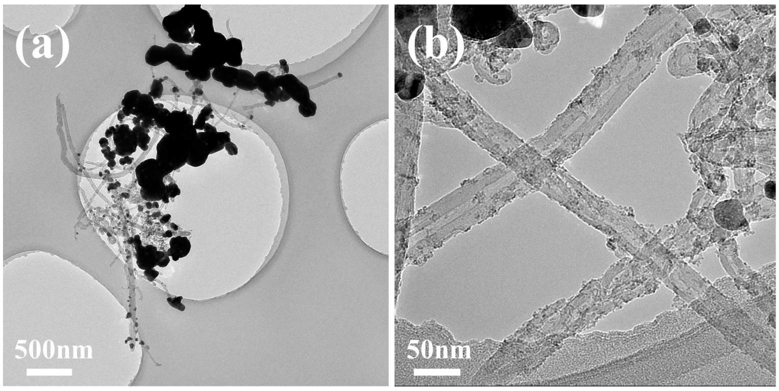


**Figure S1.** TEM images of Ag-CNT powders prepared by CP at different magnifications.

## 2. Grain size of pure Cu, CNTs/Cu and Ag-CNTs/Cu composites

The optical microscope (OM) images of pure Cu, CNTs/Cu and the 0.9 vol.% Ag-CNTs/Cu composite after grinding, polishing and etching as well as their average grain size are shown in Figure S2a. The grain average sizes of pure Cu, CNTs/Cu and the 0.9 vol.% Ag-CNTs/Cu composite was 7.59μm, 5.72μm and 4.65μm, respectively. It could be found that the addition of CNTs had the effect of refining grains. The overall grain size of pure Cu was uniform (Figure S2a), and we could clearly see the appearance of black streaks in the CNTs/Cu composite, indicating a relatively low density (Figure S2b). The 0.9 vol.% Ag-CNTs/Cu composite did not form any obvious black aggregates, indicating that the Ag-CNTs/Cu composite had high density, and the magnification showed that the grain size was relatively uniform and small (Figure S2c).

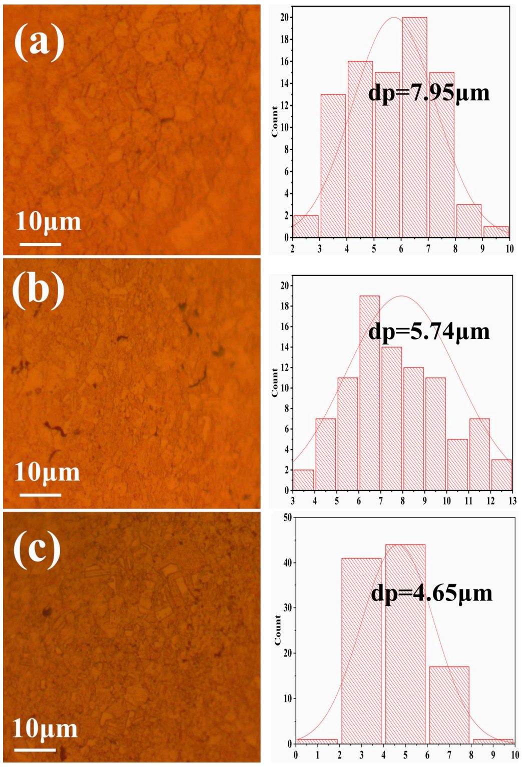


**Figure S2.** OM images and average grain size of the composites: (**a**) pure Cu; (**b**) CNTs/Cu; (**c**) 0.9 vol.% Ag-CNTs/Cu.

## Reference

[1] X.-Z. Tang, X. Li, Z. Cao, J. Yang, H. Wang, X. Pu, Z.-Z. Yu, Synthesis of graphene decorated with silver nanoparticles by simultaneous reduction of graphene oxide and silver ions with glucose, Carbon 59 (2013) 93-99.

[2] Q. Zhao, S. Tan, M. Xie, Y. Liu, J. Yi, A study on the CNTs-Ag composites prepared based on spark plasma sintering and improved electroless plating assisted by ultrasonic spray atomization, Journal of Alloys and Compounds 737 (2018) 31-38.

[3] J.-R. Choi, K.-Y. Rhee, S.-J. Park, Influence of electrolessly silver-plated multi-walled carbon nanotubes on thermal conductivity of epoxy matrix nanocomposites, Composites Part B: Engineering 80 (2015) 379-384.
